# Sleep disturbances in *SCN8A* ‐related disorders

**DOI:** 10.1002/epi4.13042

**Published:** 2024-10-03

**Authors:** Francesca Furia, Katrine M. Johannesen, Claudia M. Bonardi, Roberto Previtali, Angel Aledo‐Serrano, Massimo Mastrangelo, Jacopo Favaro, Silvia Masnada, Valentina di Micco, Jacopo Proietti, Pierangelo Veggiotti, Guido Rubboli, Gaetano Cantalupo, Kern Olofsson, Rikke S. Møller, Elena Gardella

**Affiliations:** ^1^ Department of Epilepsy Genetics and Personalized Treatment Danish Epilepsy Center Dianalund Denmark; ^2^ Institute for Regional Health Services, University of Southern Denmark Odense Denmark; ^3^ Department of Woman's and Child's Health University Hospital of Padua Padua Italy; ^4^ Pediatric Neurology Unit Buzzi Children's Hospital Milan Italy; ^5^ Department of Biomedical and Clinical Sciences University of Milan Milan Italy; ^6^ Epilepsy and Neurogenetics Unit Vithas La Milagrosa University Hospital, Vithas Hospital Group Madrid Spain; ^7^ IRCCS Policlinico San Donato Milan Italy; ^8^ Paediatric Neurology and Neurophysiology Unit University Hospital of Padua Italy; ^9^ Epilepsy and Movement Disorders Neurology Unit Bambino Gesú Children Hospital, IRCCS Rome Italy; ^10^ Innovation Biomedicine Section, Department of Engineering for Innovation Medicine University of Verona Verona Italy; ^11^ Child Neuropsychiatry Unit University Hospital of Verona Verona Italy; ^12^ Center for Research on Epilepsy in Pediatric Age (CREP) University Hospital of Verona Verona Italy; ^13^ Full Member of the European Reference Network EpiCARE Barcelona Spain; ^14^ Department of Neurology Danish Epilepsy Center Dianalund Denmark; ^15^ Institute of Clinical Medicine, University of Copenhagen Copenhagen Denmark; ^16^ Department of Pediatric Epilepsy Danish Epilepsy Center Dianalund Denmark; ^17^ Department of Neurophysiology Danish Epilepsy Center Dianalund Denmark

**Keywords:** developmental and epileptic encephalopathy (DEE), epilepsy, SCN8A, sleep disorders

## Abstract

**Objectives:**

People with neurodevelopmental disorders frequently experience sleep disturbances, negatively impairing their quality of life. We aimed to determine the prevalence and nature of sleep disturbances in patients with *SCN8A*‐related disorders.

**Methods:**

Through a collaborative network of caregivers and clinicians, we collected data about epilepsy, cognitive/motor abilities, medications, and relevant comorbidities of patients harboring a pathogenic *SCN8A* variant. The Sleep Disturbance Scale for Children (SDSC), the Children's Sleep Habits Questionnaire (CSHQ‐22‐items), and the Pediatric Daytime Sleepiness Scale (PDSS) were distributed and evaluated by factor scores and Composite Sleep Index. Video‐EEG‐polysomnographic recordings were performed.

**Results:**

We enrolled 47 patients (age range: 2–39 years), whose phenotypes ranged from *SCN8A*‐DEE to intellectual disability without epilepsy. In the majority of them (82%), sleep disturbances were reported and/or observed at the SDSC. The most frequent were difficulty in initiating and maintaining sleep (64%), followed by sleep breathing disorder (43%), sleep–wake transition disorder (34%), and daytime sleepiness (34%). Sleep disturbances were more frequent in patients with severe DEE (96%) and ongoing seizures (93%) and were more severe in patients with sleep‐related seizures. The CSHQ and PDSS confirmed difficulty in initiating and maintaining sleep. Polysomnographic recordings (9 patients/20 nights) showed an altered sleep structure in 95%, with frequent arousals, mainly not seizure related.

**Significance:**

More than 80% of patients with *SCN8A*‐related disorders presented with sleep disturbances, primarily consisting of sleep instability with difficulty of initiating and maintaining sleep. Animal studies showed sleep disturbances in *SCN8A‐* and *SCN1A*‐Dravet Syndrome mice models, suggesting a role for voltage‐gated sodium channels in the regulation of sleep. Understanding the effects of *SCN8A* dysfunction on sleep stability may guide future therapeutic efforts to alleviate this often distressing symptom also in seizure‐free *SCN8A* patients.

**Plain Language Summary:**

In this study, we analyzed sleep disturbances in patients with disorders related to a genetic mutation in the gene *SCN8A*. We found that the majority of the patients experienced sleep disturbances, mainly consisting in difficulty of initiating and maintaining sleep. Sleep disturbances were more frequent in patients with severe cognitive impairment and active epilepsy and more severe in patients with seizures during sleep.

**Key Points**

- Epilepsy and sleep have a complex bidirectional relationship, especially in people with developmental and epileptic encephalopathies (DEE).
- More than 80% of patients with *SCN8A*‐related disorders reported sleep disturbances.
- The main *SCN8A*‐related sleep disturbance was difficulty initiating/maintaining sleep (64%), followed by sleep breathing disorders (43%).
- Sleep disturbances were more often reported in patients with severe *SCN8A‐*DEE and persistent seizures.
- Polysomnographic recordings showed sleep instability with frequent awakenings, most of them not seizure related.

## 1 INTRODUCTION

*SCN8A* encodes the voltage‐gated sodium channel subunit Nav1.6, which is expressed in the brain.^1^ Neuronal hyperexcitability, seizures, and neurocognitive problems are the result of impaired Nav1.6 channel inactivation.^2^, ^3^ Pathogenic variants in the *SCN8A* gene are frequently related to epilepsy, ranging from self‐limiting epilepsies^4^, ^5^ to severe developmental and epileptic encephalopathies (DEE)^6^ often refractory to anti‐seizure medications (ASM).^7^, ^8^, ^9^ Gardella and Møller described for the first time the phenotypic spectrum of *SCN8A*‐related disorders detailing the distinguishing features of different sub‐phenotypes.^4^, ^5^, ^6^, ^10^

Epileptic seizures and sleep quality have a complex bidirectional relationship; in people with DEE, comorbidities such as intellectual disability, attention deficit, and movement disorder add complexity to this interaction.^11^, ^12^ Significant sleep disturbances are often observed in patients with DEE, causing major disruption to their quality of life.^13^ Although sleep disturbances are frequently reported in patients with genetic epilepsies, only few studies exploring this issue have been performed.^14^, ^15^, ^16^, ^17^

Studies in *SCN8A* as well as *SCN1A* mice models showed sleep disturbances, such as increased NREM and decreased REM sleep.^18^, ^19^ Additionally, the mice displayed altered circadian rhythm of corticosterone secretion, with lowered and flattened diurnal level, indicating hypofunctioning hypothalamic–pituitary–adrenal (HPA) axis, and suggesting a sodium channels' role in sleep regulation.^18^, ^19^

Our study aims to characterize the prevalence and nature of sleep disturbance in patients with different *SCN8A‐*related disorders.

## 2 METHODS

We enrolled patients with *SCN8A*‐related disorders through a network of physicians and caregivers in Europe and in the USA (14 centers). We included all patients with pathogenic *SCN8A* variants, with available electro‐clinical data, and excluded patients with *SCN8A* variants of uncertain significance and the ones who did not accept to participate in the study. We reviewed their medical history including demographic and genetic data, epilepsy features, cognitive and motor development, and relevant comorbidities. Information about seizures (types, frequency, and timing—specifically wakefulness versus sleep predominance), seizure control, and medications (anti‐seizure and sleep medications) were obtained through a semi‐structured spreadsheet.

Since the *SCN8A* phenotypic spectrum is extremely heterogeneous, using phenotypic subgroups is of pivotal importance. As we previously described based on large cohort studies,^6^, ^10^ the patients were divided into five phenotypic sub‐groups, consisting of (i) Severe DEE, (ii) Focal epilepsy of intermediate severity, (iii) Generalized epilepsy, (iv) Self‐limited familial infantile epilepsy, and (v) Neurodevelopmental disorder without epilepsy.

We defined the seizure types according to ILAE classification.^20^ We defined as “frequent seizures” when occurring from several times daily to weekly, while “rare seizures” if recurring monthly to yearly.

We distributed to caregivers the Sleep Disturbance Scale for Children (SDSC),^21^ the Children's Sleep Habits Questionnaire (22‐item version),^22^ the Pediatric Daytime Sleepiness Scale (PDSS),^23^ a sleep diary (adapted from the Sleep Council diary)^24^ (see supplementary material) to collect a description of their sleep. The SDSC has been validated in patients younger than 20 years, however, we used it also for older patients with intellectual disability, as in previous studies.^25^

When possible, we also performed 24‐h video‐EEG‐polysomnographic recordings, which have been reviewed and analyzed by two neurologists with expertise in epilepsy (EG and FF) and scored according to the AASM manual by a trained neurologist with expertise in sleep medicine (FF). The setting for video‐EEG‐polysomnographic recordings consisted of 19 EEG channels, ECG, and four EMG derivations from splenius capitis, mylohyoideus, and left and right anterior tibialis muscles. Video was recorded and reviewed for seizures and sleep movements. We analyzed the EEG and the sleep architecture including: (i) total sleep time (TST) in minutes, (ii) NREM sleep, dividedinto NREM 1 (N1), NREM 2 (N2), NREM 3 (N3) stages (total time in minutes and percentage), (iii) REM (R) stage (total time in minutes and percentage), (iv) wakefulness after sleep onset (WASO; total time in minutes), and (v) arousals rate.

According to the American Academy of Sleep Medicine manual (AASM manual),^26^ we classified modifications of the EEG frequency (with increased chin tone if in REM stage) as “arousal” if lasting 3–15 s, and as “awakening” if lasting more than 15 s. The representation of NREM3 sleep and REM sleep was considered reduced when accounting for less than 20% and 25% of TST, respectively. Conversely, the representation of NREM1 sleep was considered increased when accounting for more than 5% of TST.^26^

### 2.1 Statistical analysis

Descriptive statistics were used to determine the prevalence of patients with pathological sleep scores (Student's *t*‐test and chi‐square test).

### 2.2 Standard protocol approvals, registrations, patient consents

Ethical approval was obtained from the Danish ethical committee (SJ‐ 91: version 7). Written informed consent was provided by patients or their parents or legal guardians.

## 3 RESULTS

We enrolled 47 unrelated patients (24 males and 23 females) in age range 2–39 years (median age: 7 years), including 24 novel patients and 23 previously published. They harbored 36 different pathogenic variants (32 missense and 4 truncating), 35 recurring de novo, 3 parental inherited, and 9 unknown inheritance. An overview of the clinical features including epilepsy, neurological, behavioral, and sleep disturbances is reported in Tables 1 and 2 and Table S1.

**TABLE 1:** Overall clinical reports.

Variables	Patients (%)
Clinical features	
Epilepsy	46/47 (98)
ID	44/47 (94)
Severe/profound	29/44 (66)
Mild/moderate	15/44 (34)
Non‐verbal	31/42 (74)
Non‐ambulant	24/46 (52)
ASD	13/35 (37)
ADHD	8/35 (23)
Behavioral problems	17/35 (49)
Sleep disturbances	
Total reported	36/44 (82)
Difficulty in initiating/maintaining sleep	28/44 (64)
Snoring/other respiratory symptoms	19/44 (43)
Arousal disorders	3/44 (7)
Sleep–wake transition disorders	15/44 (34)
Daytime sleepiness	15/44 (34)
Sleep hyperhydrosis	6/44 (14)

Abbreviations: ASD, autism spectrum disorder; ADHD, attention deficit hyperactivity disorder; ID, intellectual disability.

**TABLE 2:** SDSC scale scores.

Variables	Nr of patients	Abnormal SDSC scores							
		≥1 item	DIMS	SBD	DA	SWTD	DOES	SHY	TOT
Seizure frequency									
Daily/weekly	19/46 (41%)	16/18 (89%)	7/18 (39%)	9/18 (50%)	2/18 (11%)	6/18 (33%)	7/18 (39%)	4/18 (22%)	8/18 (44%)
Monthly/yearly	12/46 (26%)	10/12 (83%)	8/12 (66%)	6/12 (50%)	1/12 (8%)	5/12 (42%)	2/12 (17%)	1/12 (8%)	7/12 (58%)
Seizure free	7/46 (15%)	**2/7 (29%)**	1/7 (14%)	1/7 (14%)	0/7 (0%)	0/7 (0%)	2/7 (29%)	0/7 (0%)	1/7 (14%)
Seizure timing									
Sleep‐related	26/30 (87%)	19/23 (83%)	11/23 (48%)	11/23 (48%)	3/23 (13%)	7/23 (30%)	5/23 (22%)	2/23 (9%)	11/23 (48%)
Not sleep‐related	4/30 (13%)	4/4 (100%)	1/4 (25%)	1/4 (25%)	0/4 (0%)	2/4 (50%)	1/4 (25%)	2/4 (50%)	1/4 (25%)
ASM									
1–2 ASM	17/46 (37%)	8/15 (53%)	4/15 (27%)	2/15 (13%)	0/15 (0%)	5/15 (33%)	3/15 (20%)	1/15 (7%)	3/15 (20%)
≥3 ASM	29/46 (63%)	26/29 (90%)	15/29 (52%)	17/29 (59%)	3/29 (10%)	8/29 (28%)	10/29 (34%)	5/29 (17%)	14/29 (48%)
Intellectual disability									
Severe/profound	29/44 (66%)	**24/28 (86%)**	15/28 (54%)	16/28 (57%)	3/28 (11%)	8/28 (29%)	10/28 (36%)	4/28 (14%)	13/28 (46%)
Mild/moderate	15/44 (34%)	8/13 (62%)	4/13 (31%)	2/13 (15%)	0/13 (0%)	3/13 (23%)	3/13 (23%)	2/13 (15%)	3/13 (23%)
Normal intellect	3/47 (6%)	2/3 (67%)	0/3 (0%)	1/3 (33%)	0/3 (0%)	2/3 (67%)	0/3 (0%)	0/3 (0%)	1/3 (33%)
Motor impairment									
Non‐ambulant	24/46 (52%)	21/23 (91%)	13/23 (57%)	13/23 (57%)	3/23 (13%)	7/23 (30%)	8/23 (35%)	3/23 (13%)	11/23 (48%)
Ambulant	22/46 (48%)	13/20 (65%)	6/20 (30%)	6/20 (30%)	0/20 (0%)	6/20 (30%)	5/20 (25%)	3/20 (15%)	6/20 (30%)
ASD/behavioral problems									
Yes	26/35 (74%)	19/25 (76%)	9/25 (36%)	10/25 (40%)	2/25 (8%)	9/25 (36%)	5/25 (20%)	3/25 (125)	9/25 (36%)
No	9/35 (26%)	6/8 (75%)	5/8 (62.5%)	4/8 (50%)	1/8 (12.5%)	1/8 (12.5%)	3/8 (37.5%)	3/8 (37.5%)	4/8 (50%)
Gastrointestinal problems									
Yes	23/33 (70%)	**20/23 (87%)**	12/23 (52%)	13/23 (57%)	2/23 (9%)	7/23 (30%)	8/23 (35%)	3/23 (13%)	12/23 (52%)
No	11/33 (33%)	7/11 (64%)	3/11 (27%)	2/11 (18%)	1/11 (9%)	3/11 (27%)	1/11 (9%)	1/11 (9%)	2/11 (18%)

*Note*: Addendum: in bold the statistical significant values (*p* < 0.005).

Abbreviations: ASD, autism spectrum disorder; ASM, anti‐seizure medications; DA, disorders of arousal; DIMS, disorders of initiating or maintaining sleep; DOES, disorders of excessive somnolence; Nr, number; SBD, sleep breathing disorders; SDSC, sleep disturbance scale for children; SHY, sleep hyperhidrosis; SWTD, sleep–wake transition disorders; TOT, total score.

### 3.1 Epilepsy features

All patients but one (98%) suffered from epilepsy, with a median age at onset of 3 months (ranging from birth to 10 years). At the latest follow‐up, 33/40 (82.5%) patients with available information had uncontrolled seizures, including tonic–clonic seizures (TCS) with generalized onset (24/33, 73%, focal onset seizures with or without impaired awareness (21/33, 64%; in two patients with evolution to bilateral TCS), tonic seizures of unknown onset (12/33, 36%), absence (9/33, 27%), myoclonic (8/33, 24%), clonic (3/33, 9%), and atonic seizures of unknown onset (1/33, 3%).

The seizure frequency ranged from yearly seizures to multiple seizures per day (Table 2); 12/33 (37%) patients with available information had daily or multiple daily seizures, 6/33 (18%) weekly or multiple weekly seizures, 8/33 (24%) monthly or multiple monthly seizures, 5/33 (15%) yearly or multiple yearly seizures, and two patients had ongoing seizures with unknown frequency. The seizure duration was usually up to 5 min (range 10–40 min).

The majority of patients with available information presented with sleep‐related seizures (26/30, 87%); 18/30 (60%) had seizures only or mainly during sleep (all motor except one), 8/30 (27%) had both motor and non‐motor seizures in sleep and wakefulness, and 4/30 (13%) only in wakefulness (Table S1).

EEG showed a slowing of the background activity (53%), and multifocal (31%) or focal (19%) interictal epileptiform discharges (IEDs). Generalized IEDs were reported in 9% of the EEGs and a sleep‐related enhancement of the IED as in DEE with Spike Wave Activation in Sleep (DEE‐SWAS) was observed in one patient.

The majority of patients (63%) were treated with 3 or more ASM, the remaining patients were either in monotherapy (17%) or were taking 2 ASM (20%); 83% were treated with sodium channel blockers and 43% were taking benzodiazepines (BZD).

### 3.2 Neurodevelopmental and behavioral features

Intellectual disability (ID) was observed in 94% of the patients, ranging from mild/moderate (34%) to severe/profound (66%). Normal cognition was reported in three patients (6%), who presented with learning difficulties or dyslexia. Most patients were non‐verbal (74%) and more than half (52%) were non‐ambulant. The majority of the patients presented with severe DEE (62%), followed by intermediate severity focal or generalized epilepsy (36%) and neurodevelopmental disorder without epilepsy (2%). Autism Spectrum Disorder (ASD) was observed in 37% of the patients, Attention Deficit Hyperactivity Disorder (ADHD) or attention deficits in 23%, and behavioral problems in 49%, mainly consisting of aggressive behavior, impulsivity, and irritability.

### 3.3 Sleep disturbances

Combining the anamnestic data with the results of all the administered questionnaires, the overall prevalence of sleep disturbances in our cohort was 82% (Table 1). The main sleep disturbances were difficulty in initiating and maintaining sleep (64%), snoring and/or other respiratory symptoms (43%), problems in the transition between sleep and wakefulness (34%), and diurnal somnolence (34%; Table 1).

### 3.4 Sleep questionnaires

**FIGURE 1:**
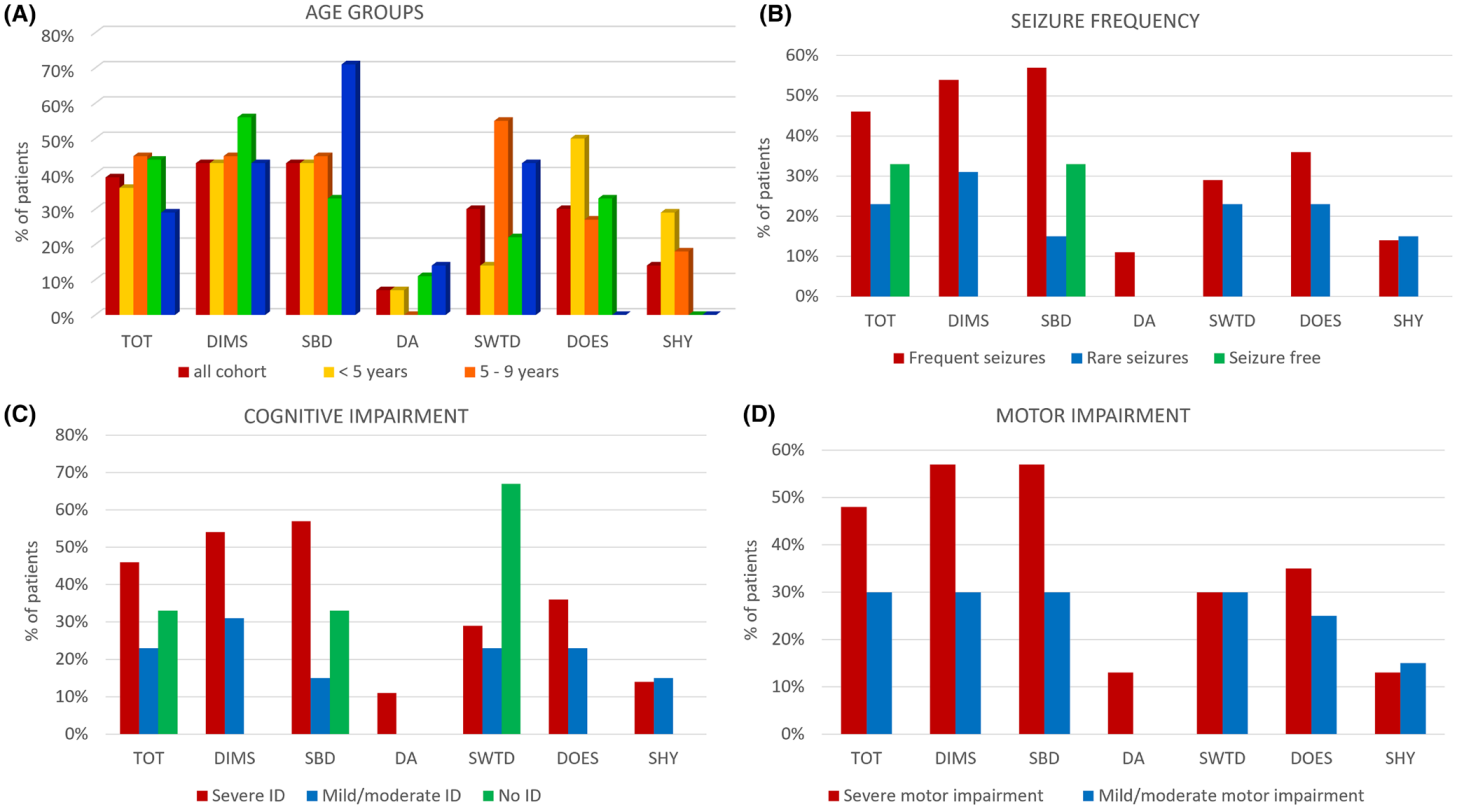
Abnormal SDSC score across different age groups and phenotypes. AGE GROUPS. The SDSC scores show DIMS as the most represented sleep disturbance in all the age groups, SBD with higher percentage in adults, DA observed in small percentages mainly in adolescents and adults, SWTD reported in all the age groups, DOES described in children and adolescents and SHY in infants and children. SEIZURE FREQUENCY. The SDSC scores reveal higher percentages of sleep disturbances in all the items in patients with persistent seizures compared to patients seizure free, without a significant difference in seizure frequency (frequent versus rare) in patients with ongoing seizures. COGNITIVE IMPAIRMENT. The SDSC scores reveal higher percentages of sleep disturbances in patients with severe ID except for SWTD that is represented in a high percentage of patients without ID. MOTOR IMPAIRMENT. The SDSC scores reveal higher percentages of sleep disturbances in patients with severe motor impairment. DA, disorders of arousal; DIMS, disorders of initiating or maintaining sleep; DOES, disorders of excessive somnolence; ID, intellectual disability; SBD, sleep breathing disorders; SDSC, sleep disturbance scale for children; SHY, sleep hyperhidrosis; SWTD, sleep–wake transition disorders; TOT, total score.

Complete SDSC questionnaires were returned by 94% of the caregivers. In 77% of the patients, at least one of the category scores was abnormal, mainly consisting of difficulty in initiating and maintaining sleep (DIMS; 43%), and in sleep breathing disorders (SBD; 43%), problems in the transition between sleep and wakefulness (SWTD; 30%) and day‐time somnolence (30%; Figure 1).

We divided the patients into 4 age groups: below 5 years (infants/toddlers; *n* = 15), between 5 and 9 years (children; *n* = 12), between 10 and 17 years (teenagers; *n* = 12), and 18 years and older (adults; *n* = 8). We observed frequent DIMS and SBD and rare DA (disorders of arousal) in all age groups. DOES (disorders of Excessive Somnolence) recurred frequently in infants/toddlers and SWTD was quite common in both children and adults (Figure 1).

Patients with ongoing seizures experienced more often (87%) sleep problems, compared to seizure‐free patients (29%; *p* < 0.005). However, the only patient without epilepsy (8 years old) also presented with sleep disturbance, consisting of SWTD.

The rate of sleep disorders was not significantly related to seizure frequency (89% in patients with “frequent seizures” versus 83% in patients with “rare seizures”), nor to the seizure distribution during sleep/wakefulness (81% vs. 100%; Table 2 and Figure 1). Sleep‐related seizure were common in patients with (73%) and without (100%) sleep disorders and the most common seizure type during sleep was motor in both groups (81% and 100% respectively; Table S1).

The severity of the most represented sleep disturbance (DIMS) was greater in patients with sleep‐related seizures (SDSC score ≥63 in 71% of patients with sleep‐related seizures versus 50% in patients with seizures not related to sleep; *p* < 0.005).

Based on sleep diary, anamnestic reports, and PSG, we observed that awakenings were long‐lasting during the nights when the patients had seizures, but a fragmented sleep was also observed in these patients during the nights without seizures (Figure 3 and Table S1).

Sleep disturbances were more frequent in patients with severe/profound cognitive and motor impairment (86% and 91% respectively), compared to patients with mild/moderate cognitive and motor impairment (62% and 65% respectively; Figure 1; *p* < 0.005). No significant differences were observed comparing patients with and without ASD and/or abnormal behavior (76% versus 75%). Patients with gastrointestinal problems, mainly consisting of dysphagia (61%, of which 57% with PEG), constipation (17%), and gastroesophageal reflux disease (GERD; 13%), were reported with more sleep disturbances (87%), as compared to patients with normal gastrointestinal function (64%; *p* < 0.005).

Completed CSHQ questionnaires were returned by 43% of the caregivers and showed a high recurrence of special needs to fall asleep, such as the presence of the parents or of some familial objects (79%), as well as the occurrence of at least one awakening during the night (53%). A smaller percentage of patients also reported daytime tiredness/sleepiness (37%), early awakening in the morning (32%), restless sleep (32%), rocking movements at falling asleep (16%), bedtime resistance (16%), bruxism (11%), and snoring (11%).

PDSS was filled out by 36% of the caregivers and its total score resulted in abnormal (>26) only in one case. The 7‐day sleep diary was completed by 62% of the caregivers and revealed a regular time schedule in all cases but one. The sleep onset latency was reported to be less than 20 min in 62% and the WASO less than 30 min in 72% of the patients.

### 3.5 Polysomnographic recordings

**FIGURE 2:**
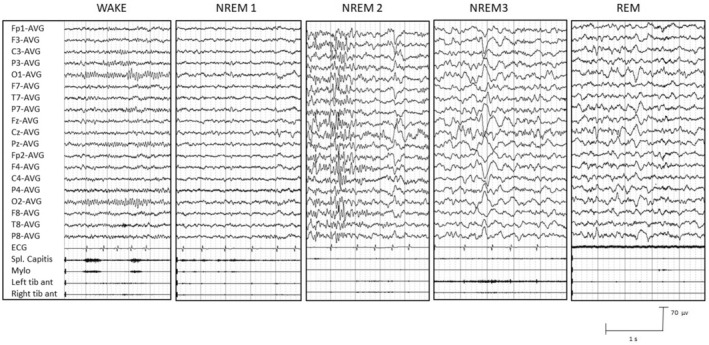
Sleep polygraphic recordings. The EEG of a patient with intermediate phenotype, characterized by focal epilepsy well controlled by the ASM and mild cognitive impairment, showing different stages of wakefulness and sleep (NREM 1, 2, 3 and REM). The background activity is represented in wakefulness state and it disappears in the transition between wakefulness and NREM 1. Vertex spikes and sleep spindles and K complex are represented in stage NREM 1 and 2 respectively. High amplitude slow delta activity is represented in NREM 3 sleep stage and faster EEG activity with lower muscular tone is present in REM stage. ECG, electrocardiogram; Mylo, mylohyoideus; NREM, non rapid eye movement; REM, rapid eye movement; Spl. Capitis, splenius capitis; Tib ant, tibialis anterior.

We analyzed 20 video‐EEG‐polysomnographic recordings in nine patients. In all patients, all sleep stages and sleep figures were represented (Figure 2). However, in all polysomnographic recordings, we observed an increased proportion of the NREM1 stage and in the majority of them a reduced proportion of NREM3 (19/20) and REM (17/20) stages, compared to what was expected for age.^25^ The mean TST was 456 min; the mean duration of NREM1 was 141 min, of NREM2 200 min, of NREM3 37 min, and of REM sleep 78 min.

**FIGURE 3:**
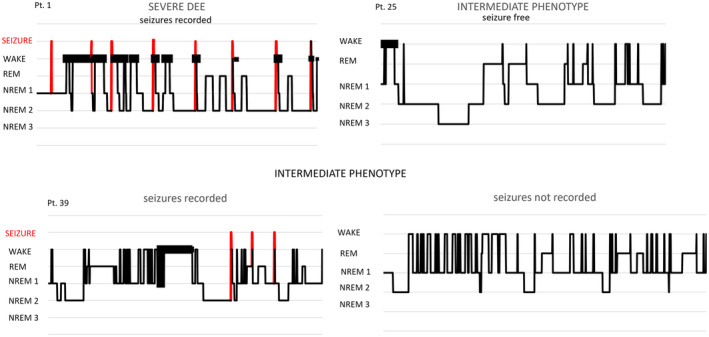
Hypnograms and sleep fragmentation. The figure shows the hypnograms of four overnight polygraphic recordings in three different patients (see the supplementary Table for clinical details). The red lines represent the time points of seizures captured during the overnight polygraphic recordings. Patient 1 has severe DEE and persistent seizures; eight seizures were recorded. The sleep is disrupted by the presence of many awakenings, only a minority of whom were seizure‐related. The percentage of REM sleep is decreased and NREM 3 sleep is not represented. Patient 25, with intermediate focal epilepsy and mild cognitive impairment, was seizure‐free at the time of recordings. The hypnogram shows the representation of all the stages of sleep with an increased percentage of WASO. Patient 39 has focal epilepsy with drug‐resistant seizures and moderate cognitive impairment. Three seizures were recorded during the first night and no seizure occurred during the second night of polysomnographic recordings. The hypnograms show sleep fragmentation with many awakenings in both registrations but higher WASO in the night with recorded seizures. DEE, development epileptic encephalopathy; NREM, non rapid eye movement; Pt, patient; REM, rapid eye movement.

In 16/20 polysomnographic recordings (9/9 patients), we observed a sleep fragmentation with increased WASO over 20 min, due to the recurrence of arousals (mean arousal index: 1.26/h) and awakenings (mean WASO: 100 min). In 8 of these recordings (5/9 patients), we also captured a total of 21 seizures, resulting in additional sleep fragmentation (Figure 3). This was consistent with our documentation of additional WASO in the polysomnographic recordings with seizures recorded, compared to the ones without seizures.

We divided the arousals/awakenings into two groups: seizure‐related and not seizure‐related. We observed that the majority of arousals/awakenings were not seizure‐related (97%). In the same subject, we compared polysomnographic recordings with and without recorded seizures: sleep fragmentation was observed in both the recordings, with and without seizures, but a long‐lasting WASO wase observed in the nights with recorded seizures (Figure 3). Interestingly, only 1/7 of these patients had pathological scores for DIMS at the SDSC.

### 3.6 Sleep medications

We obtained data about sleep medications for 21 patients; 67% were taking medicines for inducing and/or maintaining sleep, consisting of melatonin alone or in combination (79%), clonazepam (14%), niaprazine (7%), and chloral hydrate (7%). The caregivers reported the efficacy of these drugs on sleep initiation and maintenance in most cases (89%). Melatonin was reported with a good/moderate effect on difficulty in initiating and/or maintaining sleep in 71% of the patients. Chloral hydrate was described as effective in reducing night awakenings in one patient and clonazepam was effective on subjective sleep quality in two patients.

### 3.7 Parents/caregivers quality of life

Parents/caregivers of patients with sleep disturbances often reported themselves difficulty in maintaining sleep and tiredness during the day, with a bad impact on their quality of life.

## 4 DISCUSSION

Our study showed that the majority of patients with *SCN8A*‐related disorders experience sleep disturbances, mainly consisting of difficulties in initiating and maintaining sleep.

Sleep disturbances were more often reported in patients with ongoing seizures and severe/profound motor and cognitive impairment. There is a complex bi‐directional relationship between sleep, epilepsy, and developmental disorders. Many types of epilepsy have sleep‐activated seizures and interictal epileptiform discharges, with the highest preponderance reported in NREM sleep.^27^, ^28^ On the other hand, people with epilepsy can have poorer sleep quality and impaired sleep micro‐ and macro‐structure.^28^ The influence of epilepsy on sleep can be related to shared (patho)physiological mechanisms, to the effect of seizures on sleep architecture, to ongoing ASM, or a combination of all these factors.^27^, ^28^ Genetic factors can also negatively impair sleep. A number of developmental and epileptic encephalopathies with genetic etiology have been associated with specific sleep disturbances.^14^, ^16^, ^17^, ^29^, ^30^

In our cohort, we observed very frequent sleep disturbances in general, with the highest rate in patients with ongoing seizures (independently from seizure frequency), but with greater severity of the most common sleep disturbance (DIMS) in patients with sleep‐related motor seizures. This suggests a multifactorial origin of the sleep disorders, which likely results from a combined effect of epilepsy (and sleep‐related motor seizures), the developmental encephalopathy, and possibly also of the gene defect itself.

Although parents of patients with sleep disturbances may have been more willing to complete the questionnaire than those without, our overall questionnaire return rate was 65%, comparable to other studies (36%–79%).^31^, ^32^ In our cohort, 82% of patients with *SCN8A*‐related disorders were reported with sleep disturbances, which is far greater than the sleep disturbances reported in young children in the general population (31%)^33^ or in patients with epilepsy (66%).^34^ The rate and features of sleep disturbances reported in our cohort are similar to those observed in patients with Dravet syndrome (74%), mainly consisting in night awakenings (77.3%) and daytime sleepiness (40.9%).^14^ In fact, in our study 82% of patients had sleep disturbances, represented by DIMS (64%), followed by SBD (43%), SWTD and DOES (34% each).

Sleep disturbances were observed also in other genetic neurodevelopmental disorders, such as Angelman syndrome (range 20–80%),^15^ Rett syndrome (range: 80–94%),^17^ and *SYNGAP1*‐related disorders^30^ (62%). Each syndrome was characterized by specific sleep disturbances such as bedtime resistance and night awakenings in Angelman and *SYNGAP1* diseases, laughing, teeth grinding, and screaming in Rett syndrome and parasomnias and daytime sleepiness in *SYNGAP1*‐related disorders.^15^, ^16^, ^17^, ^30^

Sleep disturbances are reported as one of the major comorbidities that families coping with DEE struggle to negotiate.^35^ They could increase the likelihood of seizures due to sleep deprivation, could impact on the learning performances, and could affect the family's overall quality of life.^34^

DIMS seemed to be the most recurring sleep disturbance in patients with *SCN8A disorders*, and it was the most frequently reported problem at all ages and in all phenotypes, even if more frequent in the severe DEE. The increased propensity to wake up throughout the night represents a marker of sleep instability and might be due to altered sleep architecture in patients with *SCN8A*‐DEE, independently from the presence of seizures during the night. We documented a high WASO (mean WASO: 100 min), with an arousal index within normal ranges (mean arousal index: 1.26/h),^36^, ^37^ both in patients with and without sleep‐related seizures, suggesting sleep instability related to the *SCN8A* related disorder “per se” and not only to seizure‐related sleep disruption. However, WASO was higher during the nights when sleep‐related seizures were recorded (Figure 3), highlighting a combined influence of epilepsy on sleep. A retrospective polysomnographic study of children with *SCN1A‐*Dravet syndrome also found increased sleep instability with an increase in cyclic alternating pattern although normal arousal index.^38^

In the severe DEE group, we observed a higher percentage of all the SDSC items score, except for SHY which resulted in almost the same in the severe and intermediate phenotypes. The high prevalence of sleep breathing disorders was mainly found in individuals with severe motor impairment and might be more likely related to the neurological condition (e.g., hypotonia) than to a specific role of the gene defect on respiration. Sleep breathing disorders in our cohort were expressed especially in adults; this result is in line with the literature, where a higher prevalence is described in patients older than 30 years of age.^39^

The high percentage of SWTD in our cohort, mainly characterized by the presence of movements in the transition from wakefulness to sleep and during the night, could be overestimated and confused by the caregivers of patients with seizures and physiological hypnagogic myoclonus. A sleep PSG, unfortunately not available for the patients with reported SWTD problems, could help with the differential diagnosis.

Moderate daytime sleepiness does not appear related in our cohort to specific ASM but could be influenced by therapy (73% of the patients in poly‐therapy with 3 or more ASMs versus 27% with 1–2 ASMs).

The percentage of DA in our cohort (7%) is slightly lower than in other neurological disorders (23%),^40^ possibly related to the cognitive inability of patients with severe *SCN8A*‐DEE (62% in our cohort) to report nightmares.

The percentage of SHY in our cohort is low (14%), as reported also in other neurological disorders (7.6%).^40^

Looking at the specific sleep scales that we used for this study, we found that the SDSC was the most informative and sensitive supported by the anamnestic report from the caregivers that also added important data, followed by the CSHQ that confirmed the main features, while the PDSS did not look very sensitive.

Polysomnographic recordings on other genetic DEE showed an alteration in sleep architecture consisting of significant reduction in total sleep time, and sleep percentage, as well as significantly higher REM latency, and number of awakenings/h^41^; in Angelmann syndrome a significantly lower percentage and duration of REM sleep, and significantly higher percentage of slow waves sleep (SWS) was observed.^29^ The polysomnographic analysis in our cohort showed increase of WASO, sleep fragmentation due to arousals, and increase of light sleep (NREM1) representation, with representation of the physiological sleep figures. The high rate of awakenings and arousals was a common feature in all recordings, with and without concomitant seizures. During nights with recorded seizures, 90% of the awakenings/arousals were not seizure‐related. These results suggest an intrinsic sleep instability in *SCN8A*‐DEE. On the other hand, the fact that the WASO was more prolonged in patients with recorded seizures suggests an influence of epilepsy on sleep. Melatonin was the most used sleep medication for DIMS, effective in 71% of our cohort. The superiority of Melatonin for DIMS in DEEs have been similarly reported also in other DEEs in our experience and in the literature, followed by benzodiazepines,^14^ trazodone,^30^ and clonidine.^30^

Mouse models of *SCN1A*‐related disorders have shown that the Nav1.1 channel encoded by *SCN1A* is expressed in cells important for sleep regulation, including the *GABA*ergic neurons in the hypothalamus, thalamic reticular nucleus, and the cortex.^19^ A drug‐naive *SCN1A* Dravet syndrome mouse model demonstrated impaired sleep homeostasis secondary to the loss of Nav1.1 channels in the inhibitory forebrain *GABA*ergic neurons, implicating the gene's involvement in sleep disruption.^42^

Likewise, the *SCN8A* dysfunction may lead to sleep disruption by dysregulation of neurological sleep networks. In fact, studies on *SCN8A* mice models showed that the dysfunction of the *SCN8A* voltage gated sodium channel Nav1.6 alters sleep architecture by reducing diurnal corticosterone levels. This ends in a relative increase in the amount of NREM sleep and a decrease in REM sleep.^18^ Similarly, in our patients, we observed a reduction in REM sleep and an increase in the first stage of NREM sleep (NREM1) which represents a transition period between wakefulness and sleep. This observation, together with the high prevalence of prolonged WASO duration, confirms the hypothesis of sleep instability in subjects with *SCN8A*‐related disorders.

Several possible factors may contribute to the poor sleep quality in *SCN8A*‐related disorders. For example, refractory seizures, and multiple ASMs can increase the frequency of sleep disturbance in patients with DEE.^12^ However, the majority of patients (79%) were treated with sodium channel blockers. We did not find a significant direct effect of sodium channel blockers on sleep.

On the other hand, different comorbidities, such as autistic features, behavioral problems, and developmental delay, frequently seen in patients with DEE, may also contribute to the high rate of sleep disturbances in this population.^43^ We did not observe a significant influence of these factors in our cohort, with the exception of the presence of persistent seizures which was associated with a higher percentage of sleep disorders (87% in patients with persistent seizures vs. 29% in seizure‐free patients). This suggests that good seizure control can contribute to obtaining a better sleep quality. Melatonin, Clonazepam, and Chloral hydrate were also reported to effectively facilitate sleep initiation and/or maintenance in 89% of our patients, suggesting the importance of undertaking sleep medications in these cases.

## 5 LIMITATIONS

Even if this is the first study on sleep in *SCN8A*‐related disorders including a cohort of 47 patients with a detailed description of their sleep, this is a preliminary study, limited by the small number of patients in some of the groups analyzed. Unfortunately, due to the relative rarity of this phenotype, we were able to investigate the sleep features only in seven patients seizure‐free and in one patient with *SCN8A* disorder without epilepsy. Further studies with larger cohorts, such as polysomnographic studies in *SCN8A* patients with and without epilepsy of different age groups, are needed to better define the sleep pattern in *SCN8A*‐related disorders. Regarding the impact of patients sleep disturbances on caregivers, we did not have the possibility to ask the parents/caregivers to fill in sleep questionnaires; this can be a future step of our research.

## 6 CLINICAL RELEVANCE AND FUTURE DIRECTIONS

Sleep disturbances are a common feature of patients with *SCN8A*‐related disorders. Given the high frequency and impact of sleep disturbances in patients with *SCN8A*‐DEE, it is important to ask specifically about sleep quality and habits. Clinical evaluation, appropriate investigation, and active management are recommended especially for all patients with *SCN8A*‐related disorders who report symptoms of poor sleep. Effective management of sleep disorders and sleep related seizures is likely to improve the quality of life of the patient and the family and has the potential to optimize developmental outcome and improve seizure control.

## AUTHOR CONTRIBUTIONS

FF analyzed the data and wrote the manuscript, EG conceived and designed the study, collected and analyzed data, and wrote the manuscript, KMJ CMB, RB, AAS, MM, JF, SM, VDM, JP, PV, GR, GC, KO, RSM collected the data and reviewed the manuscript.

## CONFLICT OF INTEREST STATEMENT

None of the authors has any conflict of interest to disclose.

## ETHICAL APPROVAL

Patients gave written informed consent. All human and animal studies have been approved by the appropriate ethics committee and have therefore been performed in accordance with the ethical standards laid down in the 1964 Declaration of Helsinki and its later amendments.

## ETHICAL PUBLICATION STATEMENT

We confirm that we have read the Journal's position on issues involved in ethical publication and affirm that this report is consistent with those guidelines.

## CONSENT TO PARTICIPATE

Written informed consent was obtained from all patients in this study.

## Supporting information




Figure S1.

Table S1.
