# Supplementary material for: Sleep disturbances in SCN8A ‐related disorders
Source: Epilepsia Open. 2024 Oct 3;9(6):2186–97. doi: 10.1002/epi4.13042 (PMC11633700; doi:10.1002/epi4.13042)
Supplement: Supplementary file 1 — Figure S1. [file EPI4-9-2186-s001.docx]

**Supplementary figure 1:** sleep diary

| Day | 1 | 2 | 3 | 4 | 5 | 6 | 7 |
| --- | --- | --- | --- | --- | --- | --- | --- |
| How many naps did she/he have during the day or evening? If yes, for how long (in min)? |  |  |  |  |  |  |  |
| What time did she/he go to bed last night? |  |  |  |  |  |  |  |
| What time did she/he wake up this morning? |  |  |  |  |  |  |  |
| How long did it take for her/him to first fall asleep (in min)? |  |  |  |  |  |  |  |
| Did she/he fall asleep easily/after sometime/with difficulty? |  |  |  |  |  |  |  |
| How many times did she/he wake up in the night? |  |  |  |  |  |  |  |
| How long was she/he awake during the night in total? |  |  |  |  |  |  |  |
| How many seizures dis she/he experience during the night? |  |  |  |  |  |  |  |
| Any other note |  |  |  |  |  |  |  |
