# Supplementary material for: Sleep disturbances in SCN8A ‐related disorders
Source: Epilepsia Open. 2024 Oct 3;9(6):2186–97. doi: 10.1002/epi4.13042 (PMC11633700; doi:10.1002/epi4.13042)
Supplement: Supplementary file 2 — Table S1. [file EPI4-9-2186-s002.docx]

|  | **Supplementary table 1:** Clinical characteristics of patients | | | | | | | | | | |
| --- | --- | --- | --- | --- | --- | --- | --- | --- | --- | --- | --- |
|  |  | | | | | | | | | | |
| **Pt.** | | **sex / age *(years)*** | **SCN8A variant**  ***(inheritance / functional effect)*** | ***SCN8A***  **phenotype** | **Seizures** | | | **ASM and sleep medicines** | **Cognitive and motor assessment** | **Abnormal SDSC scores *(t-score)*** |  |
|  |  |  |  |  | **Type** | **Timing** | **Frequency** |  |  |  |  |
| 1 | | F/10.6 | c.2879T>A  p.(Val960Asp)  (*de novo* / NA) | Severe DEE | **My**, Fo | S | Weekly | TPM, OXC | Severe ID, non-verbal, non-ambulant, dystonic cerebral palsy, abnormal behavior, PEG | DIMS |  |
| 2 | | F/21 | c.5615G>T p.(Arg1872Leu)  (*de novo* / GOF) | Severe DEE | **BTC** | Unk | Unk | CBZ, CLB, PB, PHT, TPM, FBM, KD, VNS | Severe ID, non-verbal, non-ambulant, quadriplegia | SBD |  |
| 3 | | M/5 | c.2936C>T p.(Ser979Phe)  (*de novo* / NA) | Severe DEE | **BTC** | Unk | Multiple/day | LEV, ZNS, OXC,  CNZ, CLB, TPM | Severe ID, non-verbal, non-ambulant, PEG | DIMS, SBD, SWTD, DOES, TOT |  |
| 4 | | F/16 | c.3914G>C p.(Arg1305Thr)  (unk / NA) | Intermed nfc | Sz free | | | LEV, OXC, LTG,  CBD, VPA | Mild ID, verbal, ambulant, poor fine motor skills, tremor, abnormal behavior | DIMS, SBD, DOES, TOT |  |
| 5 | | F/7 | c. 802A>C p.(Ile268Leu)  (*de novo* / NA) | Intermed FE | **To**, Fo | W | Daily | OXC, LTG, KD,  LCM, LEV | Mild ID, verbal, ambulant, poor fine motor skills, poor balance, ADHD | SHY |  |
| 6 | | F/4 | c.2603T>C p.(Ile868Thr)  (*de novo* / NA) | Severe DEE | Fo, Fo to **BTC** | W | Unk | LEV, VPA, CLB,  PB, KD | Severe ID, non-verbal, non-ambulant, PEG, CVI | DIMS, DOES |  |
| 7 | | M/3 | c.4883 T>G p.(Leu1628Trp)  (*de novo* / NA) | Severe DEE | **BTC**, Fo | Unk | Yearly | LEV, PB, CBZ,  PHT, OXC | Severe ID, non-verbal, ambulant, motor delay, poor fine motor skills, ataxia | DIMS, SBD, SWTD, DOES, SHY, TOT |  |
| 8 | | M/5 | c.4850 G>A p.(Arg1617Gln)  (*de novo* / GOF) | Intermed nfc | **BTC** | Unk | Unk | LEV, OXC | Moderate ID, non-verbal, ambulant, motor delay, ataxia, ASD | DIMS, SBD, SWTD, DOES, SHY, TOT |  |
| 9 | | F/2 | c.2549G>A p.(Arg850Gln )  (*de novo* / GOF) | Severe DEE | Fo, **BTC, Sp** | Unk | Unk | CLB, VGB, PHT, TPM, LEV | Severe ID, non-verbal, non-ambulant, CVI, PEG | SBD |  |
| 10 | | M/2 | c.394T>C p.(Ser132Pro)  (*paternal* / NA) | Intermed FE | Sz free | | | CBZ | Moderate ID, non-verbal, ambulant | DOES |  |
| 11 | | M/3 | c.5615C>T p.(Arg.1872Gln )  (unk / GOF) | Severe DEE | Fo, **BTC**, Ab | Unk | Multiple/day | OXC, LTG, VPA, TPM, CLB | Severe ID, non-verbal, ambulant, ASD, abnormal behavior, GERD | DIMS, SBD, DOES, TOT |  |
| 12 | | M/10 | c.324delC p.(Thr109Argfs*7)  (*de novo* / LOF) | Severe DEE | Fo | S, W | Daily | BRV, CLB, ZNS,  FEN, MLT | Severe ID, non-verbal, non-ambulant, ataxia, athetosis, ASD, abnormal behavior, CVI | DIMS, SWTD, TOT |  |
| 13 | | F/32 | c.4850G>A p.(Arg1617Gln)  (*de novo* / GOF) | Severe DEE | Fo, **BTC**, Ab, **My** | S | Monthly | OXC, PB, VPA  (CNZ if agitation) | Severe ID, non-verbal, non-ambulant, spastic dystonic tetraparesis, ASD, abnormal behavior, PEG, CVI | DIMS, SBD, DA, SWTD, TOT |  |
| 14 | | M/23 | c.4762T >G p.(Phe1588Val)  (*de novo* / NA) | Severe DEE | **My, BTC** | S | Multiple/month | RUF, FBM, VPA, MLT, teanina, magnesium and passiflora | Severe ID, non-verbal, non-ambulant, spastic dystonic tetraparesis, choreoathetosis, dysphagia, CVI | DIMS, SBD, SWTD, TOT |  |
| 15 | | M/17 | c. 5292C>G p.(Ile1764Met)  (unk / NA) | Severe DEE | **To, BTC** | S, **W** | Multiple/month | PB, VPA, LTG | Severe ID, non-verbal, non-ambulant, dystonic quadriplegia, dysphagia, CVI | DIMS, DOES, TOT |  |
| 16 | | M/5 | c.5614C>T  p.(Arg1872Trp)  (*de novo* / GOF) | Severe DEE | Fo, **BTC, To** | S | 2/year | PHT, TPM, VPA,  CLB / MDZ as rescue | Severe ID, non-verbal, non-ambulant, dysphagia | DIMS, SBD, SWTD, TOT |  |
| 17 | | M/11 | c.5614C>T  p.(Arg1872Trp)  (unk / GOF) | Severe DEE | **BTC, To,** Fo, **My** | W | Multiple/month | VPA, LCM, CLB,  CBD, VNS, MLT | Severe ID, non-verbal, non-ambulant, chorea, PEG, CVI | DIMS |  |
| 18 | | M/2 | c.5614C>T  p.(Arg1872Trp)  (unk / GOF) | Severe DEE | **To**, Fo, **BTC, My** | **S**, W | Multiple/week | OXC, TPM, PB | Severe ID, non-verbal, non-ambulant, abnormal behavior, regurgitation | None |  |
| 19 | | M/24 | c.1201T>C p.(Tyr401His)  (*de novo* / NA) | Severe DEE | Fo, **Cl, BTC** | S | Multiple/month | VPA, PRM, ETS, Niaprazine | Severe ID, non-verbal, non-ambulant, spastic dystonic tetraparesis, ASD, ADHD, abnormal behavior, drooling | SBD |  |
| 20 | | F/3 | c.2549G>A p.(Arg850Gln)  (*maternal* / GOF) | Severe DEE | **BTC, To**, Fo, **My** | S, **W** | Multiple/month | PB, VGB, CNZ | Severe ID, non-verbal, non-ambulant, intestinal problems | DOES |  |
| 21 | | F/2 | c.5615G>T p.(Arg1872Leu)  (*de novo* / GOF) | Intermed  FE | **To, BTC**, At | Unk | Unk | LEV, OXC, PHT,  KD | Moderate ID, non-verbal, non-ambulant, choreoathetosis | None |  |
| 22 | | M/4 | c.4435A>G p.(Ile1479Val)  (*de novo* / NA) | Intermed FE | Fo to **BTC** | Unk | Unk | LEV, PHT, OXC,  CLB | Moderate ID, non-verbal, non-ambulant, dystonia, abnormal behavior | DIMS |  |
| 23 | | M/7 | c.4423G>A p.Gly1475Arg  (unk / GOF) | Severe DEE | Sz free | | | OXC, BRV | Severe ID, non-verbal, ambulant, ataxia, tremor | None |  |
| 24 | | F/8 | c. 2672T>G p.(Val891Gly)  (*de novo* / NA) | NDD | No epilepsy | | | CBZ  (for paroxysmal movement disorders) | Normal cognition with executive deficits, verbal, ambulant, ataxia, tremor, poor fine motor skills, abnormal behavior | SWTD |  |
| 25 | | F/12 | c.4764C>G p.(Phe1588Leu)  (*de novo* / NA) | Intermed nfc | Sz free | | | CBZ | Mild ID, language and motor delay, ASD | None |  |
| 26 | | F/4 | c.4423G>A p.(Arg1475Gly)  (*de novo* / GOF) | Intermed FE | Fo to **BTC** | S | Multiple/day | OXC | Normal cognition, poor balance | None |  |
| 27 | | M/13 | c.4850G>A p.(Arg1617Gln)  (*de novo* / GOF) | Intermed nfc | Sz free | | | LTG | Moderate ID, non-verbal, ASD | None |  |
| 28 | | M/8 | c.2921C>G p.(Ala974Gly)  (*de novo* / NA) | Severe DEE | Fo, **BTC**, Ab, **To, Cl** | S, W | Multiple/week | PB, PHT, LEV,  CLB, MLT | Severe ID, non-verbal, non-ambulant | SBD |  |
| 29 | | M/13 | c.1246G>A p.(Glu416Lys)  (*de novo* / NA) | Severe DEE | Fo, **BTC, To** | **S**, W | Weekly | CBZ, VPA, CLB,  MLT | Severe ID, ASD, abnormal behavior | SBD |  |
| 30 | | F/16 | c.2620G>A p.(Ala874Trp)  (*de novo* / GOF) | Severe DEE | **BTC** | S, W | Multiple/week | ZNS, CLB, FEN,  CEN, MLT | Severe ID, language delay, non-ambulant, ataxia, abnormal behavior | DA |  |
| 31 | | F/6 | c.4423G>A p.(Gly1475Arg)  (*de novo* / GOF) | Intermed FE | **BTC** | S, W | Yearly | CBZ, CLB | Moderate ID, non-verbal, constipation | None |  |
| 32 | | M/18 | c.2942G>C p.(Ser981Thr)  (*de novo* / NA) | Severe DEE | Ab, **To, BTC** | S, W | Multiple/year | VPA, OXC | Severe ID, ASD | None |  |
| 33 | | F/4 | c.669G>C p.(Arg223Ser)  (*de novo* / NA) | Severe DEE | Fo, Ab, **To, My** | S, W | Multiple/day | OXC, TPM, CLB, CBD, GBP (DZP and Paraldahyde as rescue), Chloral hydrate, MLT | Severe ID; non-verbal, non-ambulant, PEG | DIMS, SBD, DA, SHY, TOT |  |
| 34 | | M/6 | c.4378A>G p.(Ile1460Val)  (*de novo* / NA) | Intermed GE | Ab, **BTC** | S, W | Monthly | OXC, MLT | Moderate ID, ASD, constipation | DIMS, TOT |  |
| 35 | | F/13 | c.1078_1079delinsGC p.(Phe360Ala)  (unk / presumed LOF) | Severe DEE | **BTC** | S | Multiple/day | VPA, LTG, LCM,  MLT | Severe ID, non-verbal, non-ambulant, constipation, dysphagia | SBD, DOES, TOT |  |
| 36 | | M/4 | c.4384G>A p.(Val1462Ile)  (*de novo* / NA) | Severe DEE | Ab | W | Daily | LCM, CLB, PB,  PHT, CNZ | Severe ID, non-ambulant, ADHD, PEG | SBD, SWTD, DOES, SHY, TOT |  |
| 37 | | F/13 | c.1874G>T p.(Arg625Leu)  (*de novo* / NA) | Intermed GE | Sz free | | | BRV, VPA | Severe ID, abnormal behavior | None |  |
| 38 | | M/6 | c.1229_1237delTGGCCATGG p.(Val10_Met412 del)  (*de novo* / presumed LOF) | Intermed FE | Ab | W | Multiple/day | LTG, TPM, MLT | Moderate ID, ataxia, ADHD | SWTD |  |
| 39 | | M/33 | c.836C>A p.(Arg28His)  (unk / NA) | Intermed FE | FAS, **BTC** | S, W | Multiple/month | VPA, LTG, PHT,  CNZ | Normal cognition with dyslexia, ADHD, abnormal behavior | SBD, SWTD, TOT |  |
| 40 | | F/3 | c.5614C>T p.(Arg1872Trp)  (*de novo* / GOF) | Severe DEE | Fo, **BTC**, At | S | Daily | OXC, LCM, CNZ, PHT, TPM, CBD, MDZ, MLT | Non-ambulant, PEG, constipation, GERD | DIMS, SBD, DOES, SHY, TOT |  |
| 41 | | F/7 | c.2620G>A p.(Ala874Thr)  (*de novo* / GOF) | Severe DEE | Fo, Fo to **BTC, To, Cl** | S | Multiple/week | OXC | Severe ID, non-verbal, non-ambulant, dystonia, athetosis, poor fine motor skills, ADHD, abnormal behavior | DIMS, SBD, SWTD, DOES, TOT |  |
| 42 | | F/32 | c.4850G>A p.Arg1617Gln  (*paternal* / GOF) | Severe DEE | **BTC** | S | Yearly | CBZ, VPA, CLB,  FEN | Severe ID | DIMS |  |
| 43 | | F/10 | c.3794G>A p.(Cys1265Tyr)  (*de novo* / NA) | Intermed FE | FIAS | **S**, W | Daily | VPA, ETS | Moderate ID, language and motor delay, ADHD | SWTD |  |
| 44 | | F/3 | c.4594A>T p.(Ile1532Phe )  (*de novo* / NA) | Severe DEE | **BTC** | S | Unk | NA | Severe ID, non-verbal, non-ambulant, dyskinesia | NA |  |
| 45 | | M/8 | c.5458C>T p.(Arg1820*)  (*de novo* / LOF) | Intermed FE | **My**, Fo, **BTC** | S | Unk | LTG | Mild ID, language delay, ataxia | NA |  |
| 46 | | M/39 | c.437G>A p.(Cys146Tyr)  (unk / NA) | Intermed GE | Ab, **BTC**, Fo | S | Daily | LTG, ETS | Moderate ID, language delay, ataxia, tremor, ASD | NA |  |
| 47 | | M/2 | c.4948G>A p.Ala1650Thr  (*de novo* / NA) | Severe DEE | Sz free | | | PHT, TPM, KD | Severe ID, clonus, cervical dystonia, hypotonia, dysphagia | None |  |
|  |  | | | | | | | | | | |
| Ab: absences; ADHD: attention deficit and hyperactivity disorder; AEDs: antiepileptic drugs; ASD: autism spectrum disorder; At: atonic seizure; BRV: brivaracetam; BTC: bilateral tonic-clonic seizures; CBD: cannabidiol; CBZ: Carbamazepine; Cl: clonic seizure; CLB: clobazam; CNZ: clonazepam; CVI: cortical visual impairment; DA: disorders of arousal; DIMS: disorders of initiating or maintaining sleep; DOES: disorders of excessive somnolence; DZP: diazepam; ESM: ethosuximide; F: female; FAS: focal aware seizure; FBM: felbamate; FE: focal epilepsy; FFA: fenfluramine; FIAS: focal impaired awareness seizure; Fo: focal seizures; Fo to BTC: focal to bilateral tonic-clonic seizure; GERD: gastroesophageal reflux disease; ID: Intellectual Disability; Interm: intermediate; KD: ketogenic diet; LCM: lacosamide; LEV: levetiracetam; LMT: lamotrigine; M: male; MLT: melatonin; MDZ: midazolam; My: myoclonic seizures; NA: not available; NDD: Neurodevelopmental disorders without epilepsy; nfc: not further classified; OXC: oxcarbazepine; PEG: percutaneous endoscopic gastrostomy; PB: phenobarbital; PHT: phenytoin; Pt: patient; RUF: rufinamide; S: sleep; SBD: sleep breathing disorders; SDSC: sleep disturbance scale for children; SHY: sleep hyperhidrosis; Sp: epileptic spasms; STP: stiripentol; SWTD: sleep–wake transition disorders; Sz: seizure; To: tonic seizure; tot: total SDSC score.; TPM: topiramate; Unk: unknown; VGV: vigabatrin; VNS: vagus nerve stimulation; VPA: valproate; ZNS: zonisamide; W: wakefulness  **In the column seizures type:** in bold motor seizure types  **In the column seizures timing:** in bold the predominant timing when both represented | | | | | | | | | | | |
